# Dynamic changes in and time sequence of ultraviolet B-induced apoptosis in rat corneal epithelial cells

**DOI:** 10.5935/0004-2749.20220042

**Published:** 2025-08-21

**Authors:** Shaobo Du, Jiande Li, Linchi Chen, Zhanyu Niu, Lan Gao

**Affiliations:** 1 School of Life Sciences, Lanzhou University, Lanzhou 730000, China; 2 School of Stomatology, Lanzhou University, Lanzhou 730000, China

**Keywords:** Ultraviolet irradiation, Radiation, Epithelium, corneal, Epithelial cell, Cell survival, Apoptosis, Rat, Irradiação com ultravioleta B, Radiação, Epitélio anterior, Célula epitelial, Sobrevivência celular, Apoptose, Ratos

## Abstract

**Purpose:**

To systematically examine the dynamic changes and time sequence in corneal epithelial cell apoptosis after excessive ultraviolet B irradiation.

**Methods:**

Ultraviolet B (144 mJ/cm^2^) was used to irradiate rat corneal epithelial cells for 2 h. Cell morphology was observed on differential interference contrast microscopy, and the numbers of the different kinds of apoptotic cells were counted using the ImageJ software. Cell viability was measured with the 3-(4,5-dimethyl-2-thiazolyl)-2,5- diphenyl-2-H-tetrazolium bromide method. Cell apoptotic rate and loss of mitochondrial membrane potential were detected using flow cytometric analyses. The expression levels of 3 apoptotic genes were measured with real-time quantitative polymerase chain reaction at different time points within 0-24 h after irradiation.

**Results:**

After 144-mJ/cm^2^ ultraviolet B irradiation for 2 h, the expression levels of caspase-8 and *Bax* were highest at 0 h; furthermore, the mitochondrial membrane potential decreased at 0 h and remained constant for 6 h in a subsequent culture. At 6 h, caspase-3 was activated. The decrease in cell viability and increase in apoptotic rate peaked at 6 h. The caspase-3 expression level decreased within 12-24 h, which led to a decline in apoptotic rate and change in apoptotic stage.

**Conclusions:**

The corneal epithelial cells exhibited rapid apoptosis after ultraviolet B irradiation, which was associated with both extrinsic and intrinsic pathways.

## Abstract (pt)

**Objetivos:**

Explorar sistematicamente as mudanças dinâmicas e a sequência temporal no processo de apoptose de células epiteliais corneanas após excesso de irradiação com ultravioleta B.

**Métodos:**

A radiação ultravioleta B (144 mJ/cm^2^) foi utilizada para irradiar células epiteliais da córnea de rato durante 2h. A morfologia celular foi observada por meio de microscópio de contraste de interferência diferencial, e os números de diferentes tipos de células apoptóticas foram contados e registrados pelo software ImageJ. A viabilidade celular foi medida pelo método brometo de 3- (4, 5-dimetil-2-tiazolil) -2, 5-difenil-2-H-tetrazólio. A taxa apoptótica celular e a perda do potencial da membrana mitocondrial foram detectadas por meio de análises citométricas de fluxo. Os níveis de expressão de três genes apoptóticos foram medidos por reação em cadeia da polimerase quantitativa em tempo real em diferentes momentos dentro de 0-24 h após a irradiação.

**Resultados:**

Após 144 mJ/cm^2^ de irradiação com ultravioleta B por 2h, os níveis de expressão de caspase-8 e *Bax* foram maiores em 0h; o potencial da membrana mitocondrial diminuiu a 0h e permaneceu constante por 6h na cultura subsequente. Às 6h, a caspase-3 foi ativada. A diminuição da viabilidade celular e o aumento da taxa apoptótica atingiu o pico em 6h. A expressão de caspase-3 diminuiu dentro de 12 - 24 h, levando a um declínio na taxa apoptótica e alteração no estágio apoptótico.

**Conclusões:**

As células epiteliais da córnea apresentaram uma apoptose rápida após excesso de irradiação com ultravioleta B, e esse processo foi associado tanto à via extrínseca como à via intrínseca.

## INTRODUCTION

Solar ultraviolet (UV) irradiation is the main causative factor of photocarcinogenesis, photoaging, and phototoxicity^(1)^ and can induce apoptosis in keratinocytes and epithelial cells, among others^(2,3)^. UV can be divided into three bands, namely UV A (UVA, 320-400 nm), UV B (UVB, 280-320 nm), and UV C (UVC, 200-280 nm). However, a negligible amount of UVC can reach the Earth’s surface through its absorption by the ozone layer in the upper atmosphere^(4)^. UVA, which mainly affects the skin and darkens it, plays a key role in the development of melanoma and nonmelanoma skin cancers^(5)^. UVA and UVB both cause damages to the skin and eyes, but UVB is more damaging because it can damage DNA by directly mediating the formation of thymine-thymine cyclobutane dimers^(6)^ and generating reactive oxygen species (ROS) indirectly^(7)^.

The eyes and skin are exposed to UV irradiation directly and continuously. In the eyes, the cornea is the outermost layer that is directly exposed to UV irra diation. Furthermore, the cornea is divided into five layers, namely the epithelium, Bowman’s membrane, the stroma, Descemet’s membrane, and the endothelium^(8)^. Of these, the epithelium is the outermost layer; therefore, UV irradiation first interacts with the corneal epithelium as the first barrier of this structure. Moreover, the corneal epithelium absorbs a large percentage of UV irradiation and protects the lens and retina from UV-induced damage^(9)^. However, excess UV irradiation in the eyes is known to induce apoptosis in the cornea^(3)^.

Apoptosis is the process of programmed cell death. The main apoptotic pathways are the extrinsic or death receptor pathway and the intrinsic or mitochondrial pathway, and caspase-3 is the executor of apoptosis^(10)^. Apoptosis is a mechanism known to be associated with corneal cell death after UV irradiation, and corneal cell apoptosis in response to UV is dose dependent. This process involves the mitochondrial pathway and activation of caspase-9, caspase-8, and caspase-3^(11,12)^. However, few studies have reported the dynamic changes in and time sequence of this phenomenon, and any mechanisms of UVB-induced apoptosis. The present study systematically investigated the changes in cell morphology, viability, apoptotic rates, mitochondrial membrane potential (MMP), and mRNA levels of *Bax*, caspase-8, and caspase-3 after UVB irradiation of rat corneal epithelial (RCE) cells. We aimed to examine the dynamic changes and time sequence associated with the process of RCE cell apoptosis after excessive UVB irradiation.

## METHODS

### Isolation of corneal epithelial cells

Corneal epithelial cells were isolated from adult female Wistar rats of standard body weight (purchased from the animal facility of the Medical School of Lanzhou University). The experimental protocols used in this study were approved by the animal experiment ethics committee of the School of Life Sciences of Lanzhou University. The methods used were as described in our previous article^(13)^, following the operating method of two other studies^(14,15)^, with some modification. Briefly, the eyes of the rats were washed at least three times in sterile phosphate-buffered saline (PBS) supplemented with 1% (*v*/*v*) penicillin-streptomycin (100 U/mL/100 µg/mL; Sangon Biotech, Shanghai, China). Under stereoscopic dissection microscopy (Zeiss, Oberkochen, Germany), the cornea was cut off along the corneal limbal rims, and the appendant sclera was removed with sterile ophthalmic scissors to reserve the cornea only. Then, the corneal stromata and endothelium were peeled off carefully using sterile thin-tipped surgical forceps. The residual corneal stroma was peeled off little by little until the epithelia were straticulate and transparent, which were then placed in 12-well cell culture plates with 2 mL of Dulbecco’s modified Eagle’s medium/nutrient mixture F-12 (DMEM/F-12) medium (Gibco, NY, USA) supplemented with 10% (*v*/*v*) fetal bovine serum (FBS; Biological Industries, Israel). The cell culture plates were placed in a 5% CO_2_ incubator (Sanyo, Osaka, Japan) at 37°C. Then, the primary epithelial cells proliferated from the epithelial tissue. The tissue was gently disrupted by pipetting to induce the falling off of the proliferated cells from the epithelial tissue and their uniform distribution in the culture plate. When the cells adhered to the plate and grew to complete confluence, they were passaged with trypsin into a culture bottle. To detect the purity of the RCE cells, an immunofluorescence assay was performed using an anti-cytokeratin 3+12 antibody (PL Laboratories Inc., Vancouver, Canada). Cytokeratins 3 and 12 are the specific marker proteins of the corneal epithelium, and the purity of RCE cells was approximately 80%.

### Cell culture and seeding

The passaged cells were cultured in DMEM/F-12 medium supplemented with 10% (*v*/*v*) FBS, incubated at 37°C with 5% CO_2_, and passaged using trypsin when they grew to 80%-90% confluence. The cells were digested, and a suspension was made. Then, the cells were seeded in a 35-mm Petri dish. When the cells grew to 80%-90% confluence, they were used in the experiment.

### UVB irradiation procedure

The RCE cells were exposed to UVB as described in a previous study^(16)^, with some modification. The cells were rinsed with PBS once and then covered with 900 µL of PBS in 35-mm Petri dishes. The culture dishes, with lids removed, were placed at a distance of 12 cm below the UVB treatment lamp tube (Huaqiang Electronic Company, Nanjing, China). The UVB radiant exposure irradiance was 20 mW/cm^2^ at this location, which was measured using the UV-297 probe of an UV radiation meter. On the basis of previous studies of UVB-induced apoptosis and damage in corneal epithelial cells^(17-19)^, the radiant exposure used in the present study was 144 mJ/cm^2^. The formula used to compute radiant exposure was “*H*=*t* × Eë,” where *H* is the radiant exposure in J/cm^2^, *t* is the time in seconds, and Eë is the measured irradiance in W/cm^2^. In accordance with this formula, the cells were irradiated with UVB for 120 min. The same procedure was used for the culture dishes in the shamirradiated control group but with no UVB irradiation. After irradiation, PBS was removed, and the medium was added to the dish again. The cells were cultured for corresponding time periods.

### Observation of cell morphology

The cells were irradiated with 144-mJ/cm^2^ UVB for 2 h and then cultured in the Live Cell Imaging System (Zeiss) at 37°C with 5% CO_2_ for 0, 6, and 12 h such that the experimental groups were called “I (2h) + C (0 h),” “I (2h) + C (6 h),” and “I (2h) + C (12 h).” The morphological changes in the corneal epithelial cells after irradiation at 0, 6, and 12 h were recorded using a differential interference contrast microscope (Zeiss). Then, 100 cells were chosen randomly, and the numbers of the different kinds of apoptotic cells were counted using the ImageJ software (National Institutes of Health, MD, USA).

### Cell viability assay

Cell viabilities were measured on the basis of the conversion of 3-(4,5-dimethyl-2-thiazolyl)-2,5-diphenyl-2- H-tetrazolium bromide (MTT) into formazan by viable cells, which is termed the MTT method^(20)^. Briefly, the cells were irradiated with 144 mJ/cm^2^ UVB for 2 h and then cultured for 0, 6, 12, and 24 h after irradiation. These four time-point groups were named “I (2h) + C (0 h),” “I (2h) + C (6 h),” “I (2h) + C (12 h),” and “I (2h) + C (24 h),” respectively. Subsequently, 10% MTT (5 mg/mL) was added to the medium in a 35-mm Petri dish. The cells were incubated for 4 h at 37°C in the dark, and the medium was carefully aspirated with needle tubing. Then, formazan was dissolved with 2 mL of dimethyl sulfoxide. The dish was shaken slowly for 15 min. Finally, the optical density (490 nm) value was measured using a spectrophotometer (UNICO, Shanghai, China).

### Apoptosis assay based on flow cytometry

After 144-mJ/cm^2^ UVB irradiation for 2 h, the cells were cultured for 0, 6, 12, and 24 h, and then the cellular apoptotic rate in each group was detected using flow cytometry with the annexin V-fluorescein isothiocyanate (FITC)/propidium iodide (PI) double-labeling method in accordance with the manufacturer’s protocol (BestBio, Shanghai, China). After the aforementioned treatment, the cells were collected, washed in PBS twice, and resuspended in an annexin V-FITC-binding buffer successively by adding 5 µL of annexin V-FITC staining solutions. The cells were then incubated for 15 min, followed by the addition of 10 µL of PI staining solution for 5 min at 4°C in the dark. The cells were detected with flow cytometry (LSR Fortessa; BD Biosciences, NJ, USA).

### MMP determination

Rhodamine 123 was used to detect the MMP expression^(21)^. Cells were collected at 0, 3, and 6 h after 144 mJ/cm^2^ of UVB irradiation for 2 h, resuspended in PBS, and then incubated with 1 µg/mL rhodamine 123 at 37°C for 30 min in the dark. Next, the cells were washed in PBS twice and resuspended in PBS. The fluorescence intensity in each group was monitored using a flow cytometer (BD Biosciences).

### Gene expression analysis

Gene expression analysis was performed 0, 6, 12, and 24 h after 144-mJ/cm^2^ UVB irradiation for 2 h. Specifically, cells were lysed, and total RNA was isolated using a TRIzol regent (Invitrogen, CA, USA) following the manufacturer’s instructions. Then, RNA was reversetranscribed to cDNA using a PrimeScript reagent kit (TaKaRa, Tokyo, Japan). Genomic DNA was eliminated with the gDNA eraser in the kit before reverse transcription. q-PCR was performed with the q-PCR System instrument (Agilent MX3005P, CA, USA) using a SYBR Premix Ex Taq II Kit (TaKaRa) in accordance with the manufacturer’s instructions. The mRNA expression levels of the apoptosis-related genes (caspase-8, *Bax*, and caspase-3) were analyzed using the primers shown in table 1. Glyceraldehyde-3-phosphate dehydrogenase (*GAPDH*) was used as the endogenous control gene, and the expression levels of the target genes were calculated using the 2^-∆∆Ct^ method^(22)^.

**Table 1:** Primers of detected genes

Gene	Accession number	Direction	Sequence	Product length (bp)
Caspase-8	NM_022277.1	Forward	5’-TGGGACCTGGTATATCCAGTCA-3 ’	103
		Reverse	5’-GCTCACATCATAGTTCACGCCA-3 ’	
*Bax*	NM_017059.2	Forward	5’-CCACCAAGAAGCTGAGCGA-3 ’	127
		Reverse	5’-GCTGCCACACGGAAGAAGA-3 ’	
Caspase-3	NM_012922.2	Forward	5’-TACTGCCGGAGTCTGACTGGA-3 ’	86
		Reverse	5’-TCTGTCTCAATACCGCAGTCCA-3 ’	
*GAPDH*	NM_017008.4	Forward	5’-TCACCATCTTCCAGGAGCGA-3 ’	102
		Reverse	5’-CCTTCTCCATGGTGGTGAAGA-3 ’	

*GAPDH*= Glyceraldehyde-3-phosphate dehydrogenase.

### Statistical analyses

Data are presented as mean ± standard deviation. All calculations and statistical analyses were performed using SPSS 19.0 (IBM, IL, USA). Statistical comparisons were made using one-way analysis of variance followed by the least significant difference post hoc test. The significance was set as p<0.05.

## RESULTS

### Cells undergo apoptotic morphological changes after UVB irradiation

**Figure 1:**
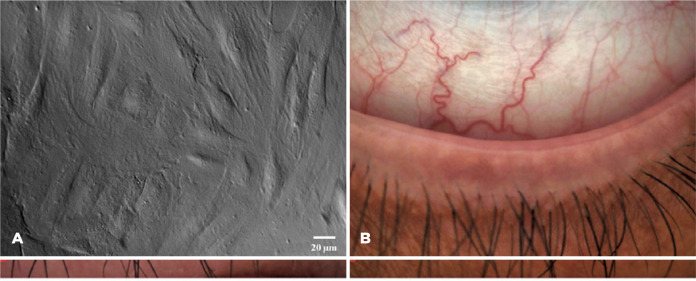
Changes in the cellular morphology of rat corneal epithelial (RCE) cells after 144-mJ/cm^2^ ultraviolet B (UVB) irradiation. The cells were irradiated with 144-mJ/cm^2^ UVB for 2 h and then cultured for 0, 6, and 12 h. The experimental groups were called “I (2h) + C (0 h),” “I (2h) + C (6 h),” and “I (2h) + C (12 h).” (A) Normal cellular morphology. (B, C, and D) Cellular morphology in the same field for the “I (2h) + C (0 h),” “I (2h) + C (6 h),” and “I (2h) + C (12 h)” groups, respectively. The morphologies of the cells at the corresponding time points 1, 2, 3, and 4 represent four typical apoptotic morphological changes after UVB irradiation, namely cell shrinkage (1), decreased cell adherence ability (2), cell membrane rupture (3), and nuclear condensation (4).

After UVB irradiation, the cells appeared to exhibit obvious apoptotic morphologies, including (1) cell shrinkage, (2) a decline in cell adherence ability, (3) cell membrane rupture, and (4) nuclear condensation. The 4 cells marked 1, 2, 3, and 4 in figure 1 represent the 4 aforementioned morphological changes. At each time point, 100 cells were chosen randomly, and the 4 types of morphological changes were recorded (Table 2). The apoptotic morphology in the “I (2h) + C (6 h)” and “I (2h) + C (12 h)” groups was more obvious than that in the “I (2h) + C (0 h)” group, and the cell membrane rupture was not recoverable.

**Table 2:** Numbers of different kinds of apoptotic cells

	Cell shrinkage and decline in adherence ability	Cell membrane rupture	Nuclear condensation
1 (2h) + C (0 h)	11.0 ± 0.5	3.3 ± 0.3	7.7 ± 0.3
1 (2h) + C (6 h)	61.0 ± 1.0	14.3 ± 0.6	29.0 ± 0.5
1 (2h) + C (12 h)	53.7 ± 0.8	14.3 ± 0.6	24.3 ± 0.3

A total of 100 cells were chosen randomly, and morphological changes were recorded. “I (2h) + C (0 h)”, “I (2h) + C (6 h)”, and “I (2h) + C (12 h)” represent cells that were irradiated with 144 mJ/cm^2^ UVB for 2 h and then cultured for 0, 6, and 12 h, respectively, after irradiation. Data are presented as mean ± standard deviation, *n*=3.

### Cell viability after UVB irradiation

**Figure 2:**
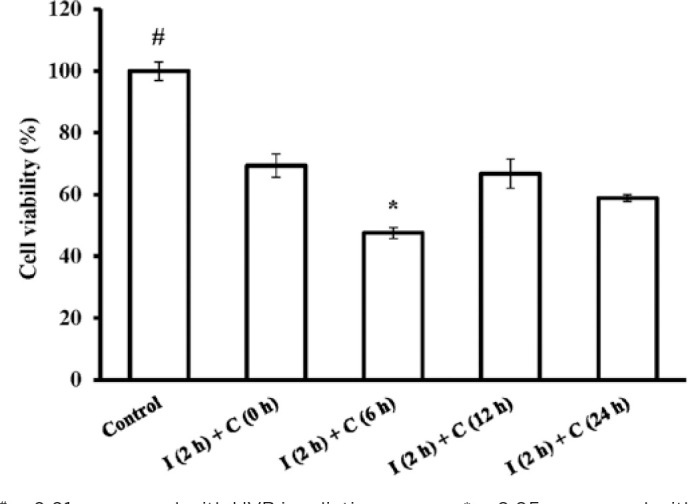
Variation in the cell viability of rat corneal epithelial (RCE) cells after 144-mJ/cm^2^ ultraviolet B (UVB) irradiation. “I (2h) + C (0 h),” “I (2h) + C (6 h),” “I (2h) + C (12 h),” and “I (2h) + C (24 h)” represent cells irradiated with 144-mJ/cm^2^ UVB for 2 h and then cultured for 0, 6, 12, and 24 h, respectively, after irradiation. Data are presented as mean ± standard deviation, *n*=3.

^#^p<0.01, compared with UVB irradiation groups. *p<0.05, compared with other irradiation groups.

Cell viability was detected at 0, 6, 12, and 24 h using the MTT method after 144 mJ/cm^2^ of UVB irradiation for 2 h. The results showed that the cell viabilities in all the UVB irradiation groups were significantly lower than that in the control group (p<0.01). Furthermore, of the UVB irradiation groups, the “I (2h) + C (6 h)” group had significantly lower cell viability than the other groups (p<0.05; Figure 2).

### Cellular apoptotic rate after UVB irradiation

**Figure 3:**
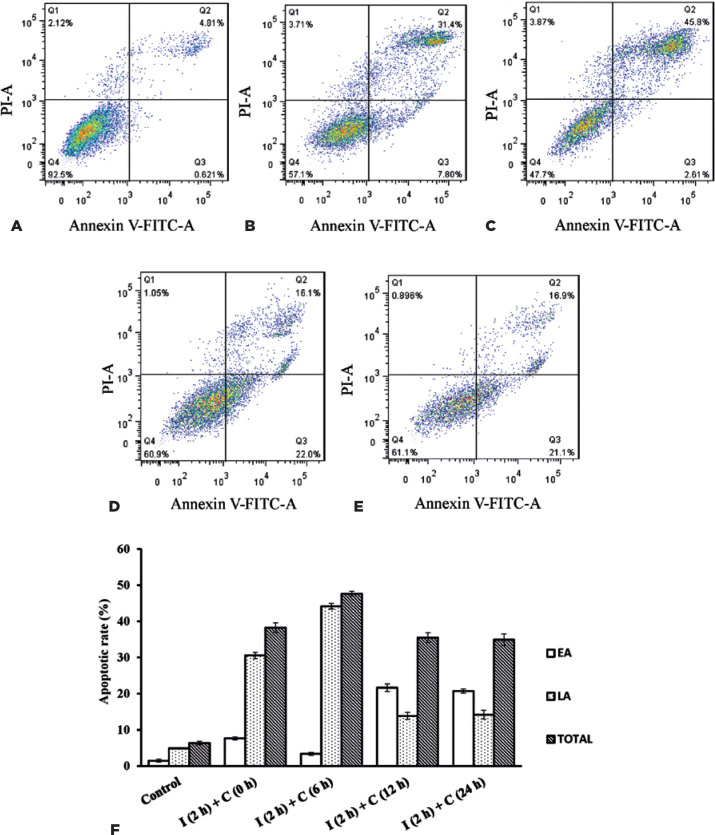
Variation in the apoptotic rate of rat corneal epithelial (RCE) cells after 144-mJ/cm^2^ ultraviolet B (UVB) irradiation within 24 h. The cells were irradiated with 144-mJ/cm^2^ of UVB for 2 h and then cultured for 0, 6, 12 h, and 24 h. The experimental groups were called “I (2 h) + C (0 h),” “I (2 h) + C (6 h),” “I (2 h) + C (12 h),” and “I (2 h) + C (24 h).” (A-E) The flow cytometry scatter plot. (A) The control group and (B-E) “I (2h) + C (0 h),” “I (2h) + C (6 h),” “I (2 h) + C (12 h),” and “I (2h) + C (24 h)” groups. Q1: Dead cells; Q2: late apoptotic cells; Q3: early apoptotic cells; and Q4: normal cells. (F) Statistical analysis of changes in the apoptotic rate displayed using a histogram.

Cellular apoptosis was quantified at 0, 6, 12, and 24 h after 144-mJ/cm^2^ UVB irradiation for 2 h (Figure 3A-3E).

EA= early cellular apoptotic rate; LA= late cellular apoptotic rate; TOTAL= total cellular apoptotic rate=(early cellular apoptotic rate) + (late cellular apoptotic rate). Data are presented as mean ± standard deviation, *n*=3.

The apoptotic rates were measured, and the values are displayed in the histogram in figure 3F. The total cellular apoptotic rate was calculated as a sum of the early and late cellular apoptotic rates. The RCE cells underwent apoptosis gradually during exposure to UVB irradiation, and the apoptotic rates showed dynamic changes within 24 h after UVB irradiation. The total apoptotic rate was highest at 6 h [I (2h) + C (0 h): 38.23% ± 1.34%; I (2h) + C (6 h): 47.61% ± 0.65%; I (2h) + C (12 h): 35.50% ± 1.33%; and I (2h) + C (24 h): 34.93% ± 1.62%] within the 24-h period after irradiation. Moreover, the apoptotic cells in the two stages underwent dynamic changes at different time points after UVB irradiation. Within 6 h after irradiation, most apoptotic cells were at the late stage of apoptosis. However, changes were more frequently observed among the early than among the late apoptotic cells within 12-24 h.

### MMP after UVB irradiation

**Figure 4:**
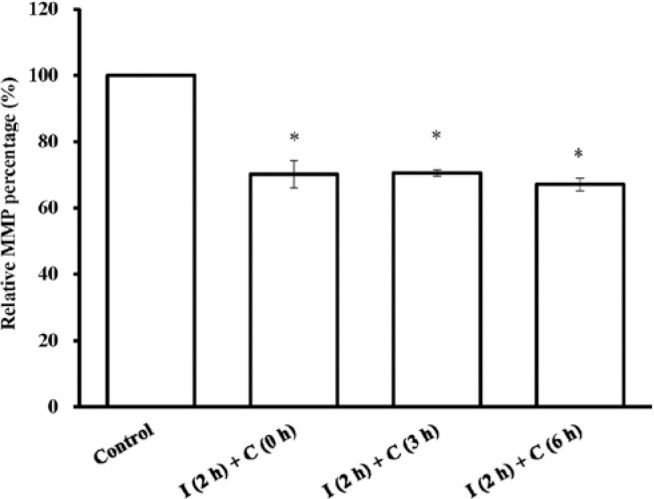
Statistical analysis of rhodamine 123 relative fluorescence intensity in rat corneal epithelial (RCE) cells after 144-mJ/cm^2^ ultraviolet B (UVB) irradiation. “I (2h) + C (0 h),” “I (2h) + C (3 h),” and “I (2h) + C (6 h)” represent cells irradiated with 144-mJ/cm^2^ UVB for 2 h and then cultured for 0, 3, and 6 h, respectively, after irradiation. Data are presented as mean ± standard deviation, *n*=3.

To address the mechanism underlying the apoptotic changes induced by UVB related to the mitochondrial pathway, MMP expression was detected as shown in figure 4. The MMP expression level was found to decrease at 0 h immediately after 144-mJ/cm^2^ UVB irradiation for 2 h and remained constant for 6 h until the highest cell apoptotic rate after irradiation.

*p<0.05, compared with the control group.

### mRNA expression levels of caspase-8, *Bax*, and caspase-3 after irradiation

**Figure 5:**
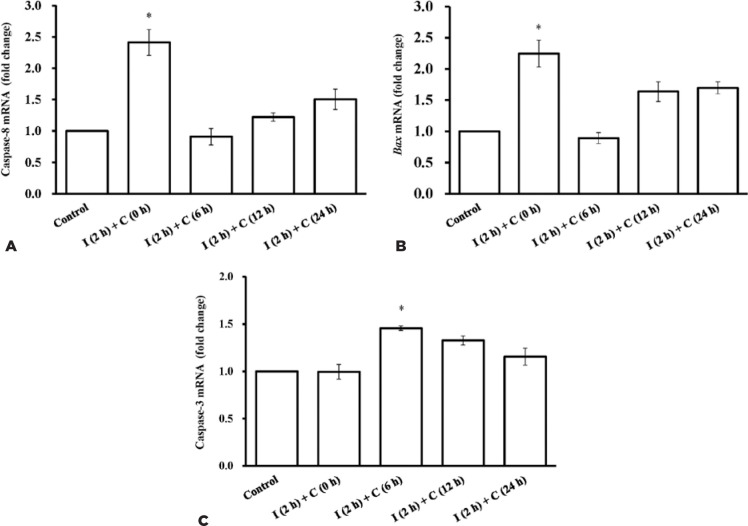
Changes in the mRNA expression levels in the apoptosis markers after ultraviolet B (UVB) irradiation. The levels of caspase-8 (A), *Bax* (B), and caspase-3 (C) in rat corneal epithelial (RCE) cells after 144 mJ/cm^2^ ultraviolet B (UVB) irradiation within 24 h are shown. “I (2h) + C (0 h),” “I (2h) + C (6 h),” “I (2h) + C (12 h),” and “I (2h) + C (24 h)” represent cells irradiated with 144-mJ/cm^2^ UVB for 2 h and then cultured for 0, 6, 12, and 24 h, respectively, after irradiation. The mRNA expression levels of caspase-8 and *Bax* were highest in the “I (2h) + C (0 h)” group, whereas that of caspase-3 peaked at 6 h after irradiation. Data are presented as mean ± standard deviation, *n*=3.

To clarify the changes in apoptotic genes in the cells after UVB irradiation, the mRNA expression levels of caspase-8, *Bax*, and capase-3 were measured after UVB irradiation. Caspase-8 and *Bax* are the genes involved in the extrinsic and intrinsic pathways of apoptosis, respectively, whereas caspase-3 is the executor of apoptosis. The results showed that the mRNA expression levels of caspase-8 (Figure 5A) and *Bax* (Figure 5B) within 24 h after UVB irradiation were highest in the “I (2h) + C (0 h)” group, whereas that of caspase-3 (Figure 5C) was highest in the “I (2h) + C (6 h)” group. We found that the apoptotic program of the RCE cells was initiated gradually during exposure to UVB irradiation, and upregulation of the proapoptotic factors was most significant 0 h after 2 h of UVB irradiation, which resulted in the upregulation subsequent cultures probably because of the effects of of the apoptotic executor 6 h after irradiation, which antiapoptotic factors. In addition, the expression level led to rapid cell apoptosis. The expression levels of proof caspase-3 decreased within 12-24 h after UVB irraapoptotic factors *Bax* and caspase-8 decreased in the diation, which led to a decline in the apoptotic rate and change in the type of apoptotic cells. These results were characterized by the cell viability and cellular apoptotic rate as mentioned earlier.

*p<0.05, compared with the other irradiation groups.

## DISCUSSION

UVB irradiation is known to damage cells and induce cell apoptosis. The relationship between UVB and the eyes has been extensively studied because the eyes are sensitive to irradiation and exposed directly to solar UV. Damage to the eyes and the variation of the viability of corneal epithelium cells have been reported to be strongly dependent on the dose received during UVB irradiation^(18,23)^. However, the detailed process of the dynamic changes after UVB irradiation of the corneal epithelium cells is still controversial and needs to be investigated further.

Previous studies reported various changes in cellular morphology after UVB irradiation. Studies on corneal epithelial cells demonstrated that the cells appeared to be separated and elongated, with decreased adhesiveness and cell adherence ability, after UVB irradiation^(11)^. The morphological changes observed after UVB exposure in other types of eye cells among rat retinal ganglion cells and human retinal pigment epithelial cells include bleb formation, vacuolation, and membrane rupture^(24)^. The previous and present results show that the morphological changes in eye cells after excess UVB irradiation usually include cell shrinkage or reduced adhesiveness, membrane rupture, and weakened cell adherence. Moreover, we found that these apoptotic morphological changes became obvious gradually within 0-6 h of culture after UVB irradiation.

The cell viability declined, and the cell apoptotic rate increased after UVB irradiation. The apoptotic index of the rodent corneal epithelium cells increased at 30 min (12.12 ± 0.63), 2 h (28.0 ± 1.3), 6 h (31.02 ± 1.53), and 24 h (25.63 ± 1.05) after UV irradiation as compared with that of the controls (5.82 ± 0.30), and this tended to increase first and then decrease in the previous research^(25)^. In the present study, we measured both cell viability and apoptotic rate, and found that the changes in these two parameters also appeared to follow the same trend. Furthermore, the present study showed that the most obvious loss (46.33% of the control group) of cell viability occurred at 6 h after UVB irradiation. Similarly to the change in cell viability, an increase in cell apoptotic rate was induced by UVB, which was also highest at 6 h after irradiation.

To clarify the in-depth dynamic changes in apoptosis induced by UVB, the expression levels of MMP and some apoptotic genes involved in extrinsic and intrinsic pathways were studied. One review reported that the mitochondria have several functions within the cell, including its key involvement in apoptotic events, and that mitochondrial function is a key mediator of UV-induced apoptosis^(26)^. In the present study, we found that 144-mJ/cm^2^ UVB caused a decrease in the MMP expression level as compared with that in the control group at 0 h immediately after 2 h of UVB irradiation, and the value remained constant for 6 h. This suggests an involvement of the mitochondrial pathway in this process. Studies have shown that UVB upregulates the expressions of apoptotic genes, including caspase-8, *Bax*, and caspase-3 dose dependently^(18,24)^. In the present study, we found that the expression levels of caspase-8 and *Bax* peaked immediately in the “I (2 h) + C (0 h)” group, whereas the expression level of caspase-3 reached a maximum 6 h after UVB irradiation. These results correspond with the upstream/downstream relationships of the three aforementioned genes. These findings also imply rapid mitochondrial response and activation of caspases after UVB irradiation.

UVB irradiation is a major ROS inducer on the ocular surface^(27)^, and 150-mJ/cm^2^ UVB was previously found to induce a 384% increase in the ROS levels in human epithelial corneal cells^(18)^. The increased ROS level is a trig ge ring event upstream of DNA damage, mitochondrial membrane depolarization, and caspase activation^(18,28)^. The present study shows that UVB induced a decrease in the MMP level and the activation of caspases in RCE cells. These findings suggest that the increase in ROS level and DNA damage, decrease in MMP level, and acti vation of caspases are involved in UVB-induced events in RCE cells. Moreover, these events occurred within a short time after irradiation.

**Figure 6:**
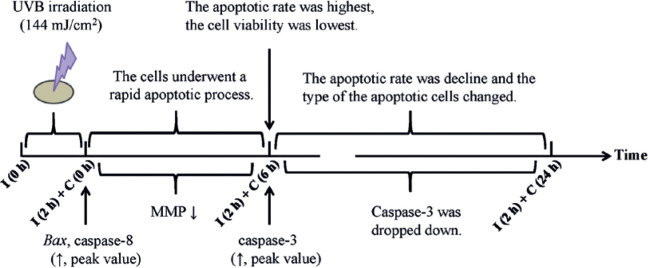
Time sequence of the ultraviolet B (UVB)-induced apoptosis of rat corneal epithelial (RCE) cells.

All of the aforementioned apoptotic factors are interrelated. One recent study has shown that caspase-8 is required for caspase-3 activation in the apoptotic extrinsic pathway and that capase-8 is a highly robust enzyme that activates caspase-3^(19)^. In the apoptotic intrinsic pathway, the Bcl-2 family of proteins governs mitochondrial membrane permeability and then induces the release of cytochrome c from the mitochondria into the cytoplasm^(10,29,30)^. In the present study, these changes in UVB-induced RCE cell apoptosis occurred in a specific temporal sequence and as a dynamic process. *Bax* and caspase-8, which encode proapoptotic factors, were upregulated at 0 h after 2 h of 144-mJ/cm^2^ UVB irradiation. Moreover, the MMP decreased and remained constant for 6 h. All these factors activated the apoptotic executor caspase-3. The cell viability declined, and cell apoptosis was most obvious at 6 h after irradiation. Furthermore, the caspase-3 expression level decreased within 12-24 h because of antiapoptotic mechanisms, which led to a decline in the apoptotic rate. A more interesting finding is that most apoptotic cells were at a late stage from 0-6 h after UVB irradiation, but the changes were more frequent in the early than in the late apoptotic cells at 12-24 h. A summary of these findings is shown in Figure 6. These results suggest that corneal cells undergo a rapid apoptotic process after excess UVB irradiation, which was found to involve both the extrinsic and intrinsic pathways. The present study provides reference values and a time window for the prevention and treatment of UVB-induced damage to corneal epithelial cells, as well as the protection of the corneal epithelial cells from UVB-induced apoptosis. In addition to the subject of the present research, the dynamic changes in limbal stem cells after UVB irradiation would also be interesting to study, which could be explored in the future.

I= UVB irradiation, C= culture after UVB irradiation.
